# Supplementary material for: CabZIP23 Integrates in CabZIP63–CaWRKY40 Cascade and Turns CabZIP63 on Mounting Pepper Immunity against Ralstonia solanacearum via Physical Interaction
Source: Int J Mol Sci. 2022 Feb 28;23(5):2656. doi: 10.3390/ijms23052656 (PMC8910381; doi:10.3390/ijms23052656)
Supplement: Supplementary file 1 [file ijms-23-02656-s001.zip › ijms-1583551-SI.pdf]

Supplemental Table

Table S1. Primers used in this study.

	Gene	Gene bank/ Accession #	Forward primer	Reverse primer
Primers used for *CabZIP23* study	***CabZIP23***[1]	XM_016684477.1	GGGGACAAGTTTGTACAAAAAAGCAGGCTTCATGGCTGACGGGGAGCTGGA	GGGGACCACTTTGTACAAGAAAGCTGGGTCTTAACAGAATAGGAACATCA
	***CabZIP23*-GFP**[2]	XM_016684477.1	GGGGACAAGTTTGTACAAAAAAGCAGGCTTCATGGCTGACGGGGAGCTGGA	GGGGACCACTTTGTACAAGAAAGCTGGGTCACAGAATAGGAACATCAATT
	***Ca bZIP23*-VIGS**[3]	XM_016684477.1	GGGGACAAGTTTGTACAAAAAAGCAGGCTTCTCGGAAGACGATAAGAACCC	GGGGACCACTTTGTACAAGAAAGCTGGGTCCCCTTCAATCCTTCCTCGTA
Primers used for pepper q-PCR analysis study	***CabZIP23*-qPCR**	XM_016684477.1	GCGTCTCTGCTACTGCTGAA	AATCTTCTTGCCCTCCAGCC
	***CaWRKY40*-qPCR**	AAX20040.1	GGTGTGGCAGATGATAGTGC	CCAGGCACAACATCCAAGT
	***CaPR1*-qPCR**	XM_016683907.1	GCCGTGAAGATGTGGGTCAATGA	TGAGTTACGCCAGACTACCTGAGTA
	***CaNPR1*-qPCR**	X61679.1	ACTTCTTCGCCGACGCCAAG	GCCAACACATTCACCAGAGCATC
	***CaDEF1*-qPCR**	AF442388	GTGAGGAAGAAGTTTGAAAGAAAGTAC	TGCACAGCACTATCATTGCATACAATTC
	***CabZIP63*-qPCR**	XP_016537747.1	ACGACATTGCCGATCAATTA	GCAAACGATGCGGTATTAGA
	***CaHSP24*-qPCR**	HM132040	GTTCGTCTAGCAGTTTGGTTCGGTTG	GTAATTTAACTAAACAGACTCTTACAACC
	***CaACTIN*-qPCR**	GQ339766	AGGGATGGGTCAAAAGGATGC	GAGACAACACCGCCTGAATAGC
Primers used for ChIP-PCR	***CabZIP23*-W-box**	XM_016684477.1	CGACACAAACTATAACTTTTAAGAACA	AAAAGCTGTTTTTGGGTTTAGAA
	***CabZIP23*-CK**	XM_016684477.1	CCTTAGCAAACCACACTCCA	CCACGCTATTTATTACAGCCATT
	***CabZIP63*-G-box-1**	XP_016537747.1	TTTTATCAAACTTTAAAAGAAGAT	ACATCTATGCTCCTATGGGATGGT
	***CaWRKY40*-C-box**	AAX20040.1	TATTCTCAAAAAATTCAATC	ATTCAAGTGTTTGTTTACAA
	***CaWRKY40*-G-box**	AAX20040.1	AACCAAGATTGTACTATAGC	AATTGCCCTTTTAAGAAGAG
	***pCaWRKY40*-CK**	AAX20040.1	TGCATGTGTTACTGTACCCA	AAATAAGATGAAGGTATACA
	***CaPR1*-G-box**	XM_016683907.1	CCAATCATCAATAGCAGATAG	GTCAAAGAATGACATGTCATG
	***CaNPR1*-G-box-1**	X61679.1	GCTATACCTTTTTACCCTATG	CATGTTAATGCATGCATGAG
	***CaNPR1*-G-box-2**	X61679.1	GACAAACGGTGGTGATTGC	CCAAGGGGACGACTGAATGAGG
	***CaHSP24*-G-box**	HM132040	CCAACTCACTATTACAGC	CTCAAGAGAAAGAAGGAGCTGC

[1]Primers used for ***CabZIP23*** full-length cloning

[2]Primers used for *35S:: **CabZIP23**-GFP* construct

[3]Primers used for TRV*::* ***CabZIP23*** construct

Supplemental Figures

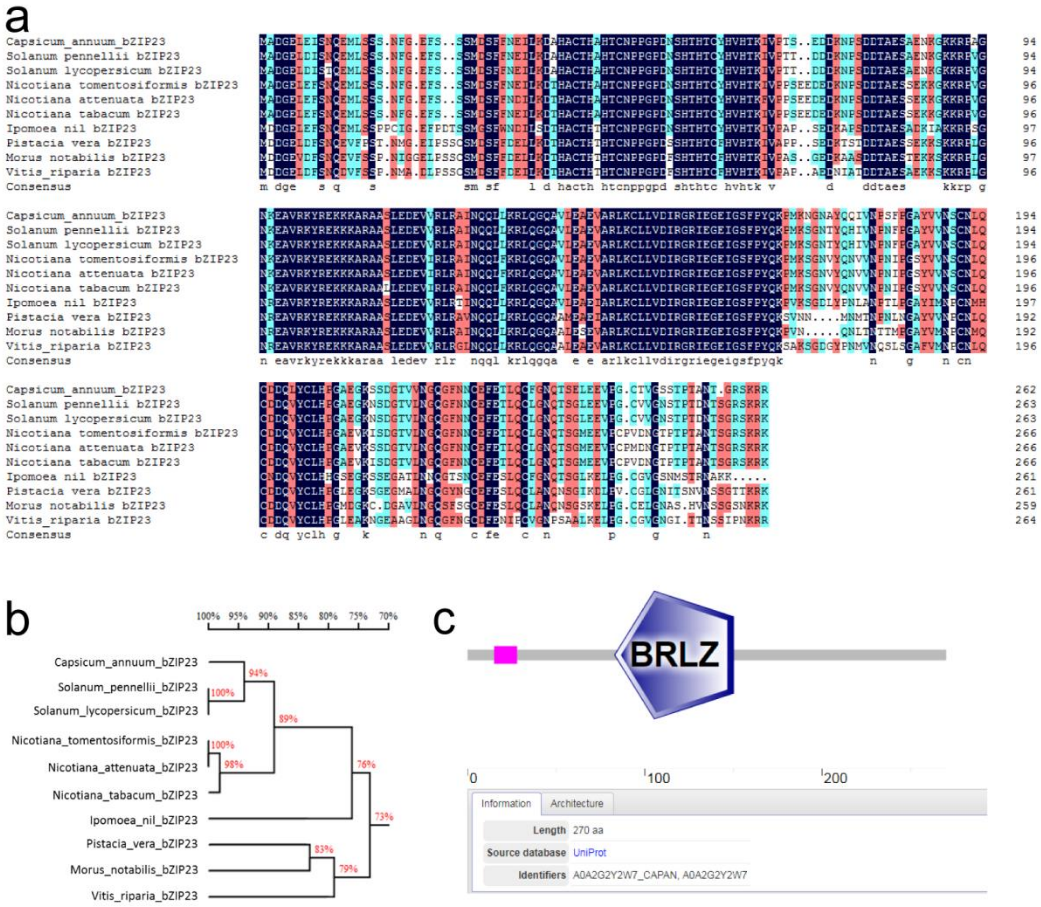

Figure S1. Analysis of amino acid sequence of pepper *CabZIP23* and the multiple alignment of CabZIP23 with its orthologues in other plant species. (**a**) Multiple alignment of amino acid sequences deduced from pepper *CabZIP23* with its orthologues from other plant species including Solanum pennellii, Solanum lycopersicum, Nicotiana tomentosiformis, Nicotiana attenuate, Nicotiana tabacum, Ipomoea nil, Pistacia vera, Morus notabilis, Vitis riparia. Blue shading, 50%-75% identity; red shading, 75%-100% identity; black shading, 100% identity. (**b**) Phylogenetic analysis of *CabZIP23* with its orthologs in other plant species. (**c**) The highly conserved BRLZ domain in *CabZIP23* amino acid sequence.

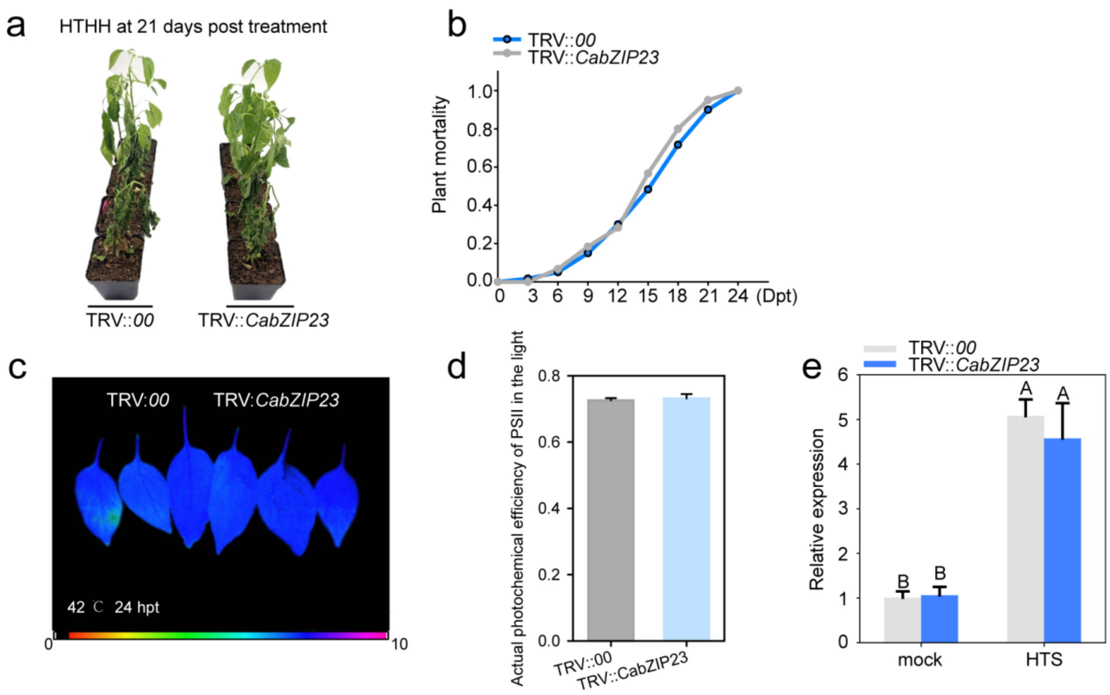

Figure S2. Effect of *CabZIP23* silencing on thermotolerance of pepper plants. (**a**) Phenotype in *CabZIP23* silenced and non-silenced pepper plants under treatment of high temperature and high humidity(HTHH). (**b**) Survival rate of pepper plants with and without *CabZIP23* silencing under HTHH treatment. (**c**) and (**d**) kinetic parameters of chlorophyll fluorescence of *CabZIP23* silenced and control pepper plants treated with HTHH, the pepper leaves were harvested at 24 hpt for chlorophyll fluorescence measurement. (**e**) Relative expression level of *CabZIP23* in *CabZIP23* silenced and non-silenced leaves under HTS by qRT-PCR. The data presented are means 6 standard error (SE) of four replicates, different capital letters indicate significant differences among means ($P < 0.01$), as calculated with Fisher's protected LSD test.

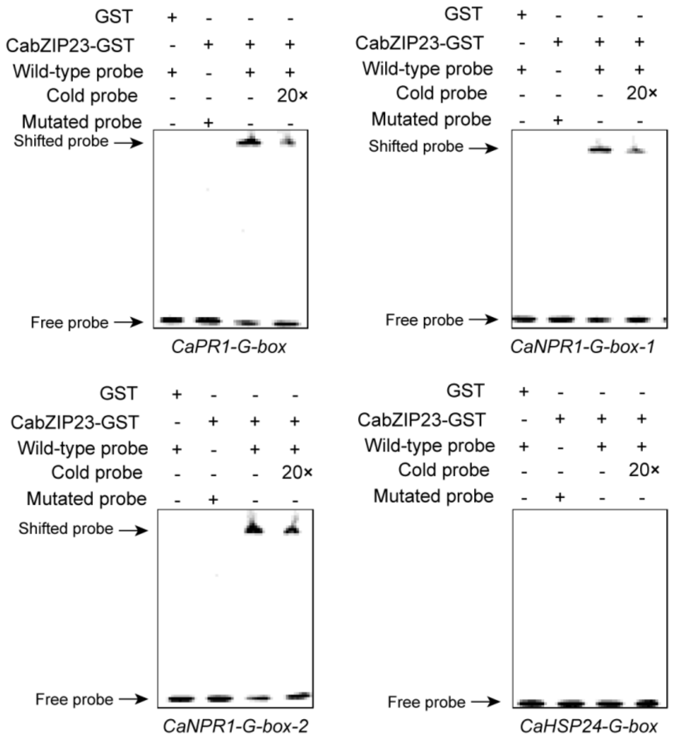

Figure S3. The binding of CabZIP23-GST to G-box ,G-box1 and G-box2 in promoters of *CaPR1*,*CaNPR1*, *CaHSP24* by EMSA. The data indicate that CabZIP23 bound the promoters of the immunity related target genes, but did not bind the promoter of *CaHSP24*.

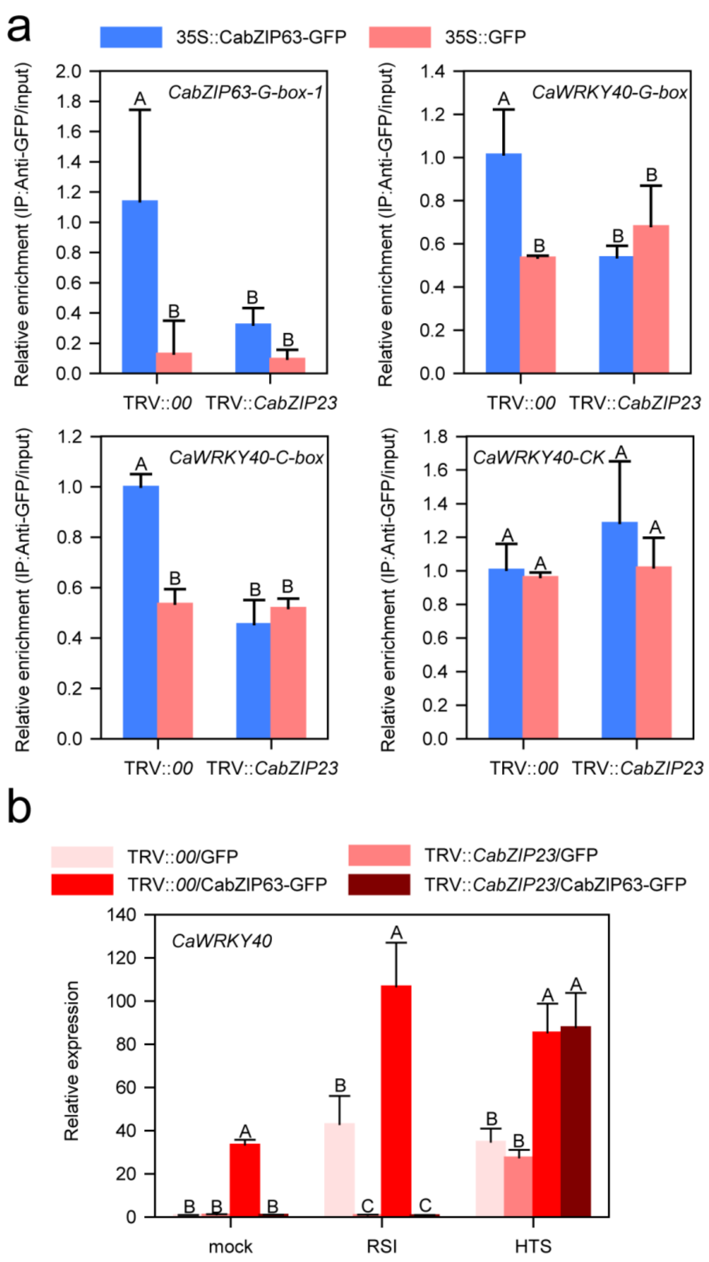

Figure S4. The effect of CabZIP23 on the regulation of CaWRKY40 expression by CabZIP63. (**a**) Detect the effect of overexpression of *CaWRKY40* on *CabZIP23* expression levels. (**b**) ChIP-qPCR was used to analyze for the binding of *CabZIP23* to 1Gbox and 2Gbox within *CabZIP63* promoters and binding of *CaWRKY40* to Wbox within *CabZIP23* promoters.

(**c**) Detection of the relative expression of *CabZIP23* in *CabZIP63* silent and non-silent leaves under RSI or HTS by qRT-PCR (**d**) qRT-PCR analysis of *CabZIP23*, *CabZIP63* and *CaWRKY40* transcript expression level in control and *CabZIP23*- silencied pepper leaves by transient overexpression of *GFP* or *CabZIP63*. The data presented are means 6 standard error (SE) of four replicates, different capital letters indicate significant differences among means ($P < 0.01$), as calculated with Fisher's protected LSD test.
